# Variations in the oral microbiome are associated with depression in young adults

**DOI:** 10.1038/s41598-021-94498-6

**Published:** 2021-07-22

**Authors:** Benjamin Wingfield, Coral Lapsley, Andrew McDowell, Georgios Miliotis, Margaret McLafferty, Siobhan M. O’Neill, Sonya Coleman, T. Martin McGinnity, Anthony J. Bjourson, Elaine K. Murray

**Affiliations:** 1 Northern Ireland Centre for Stratified Medicine, Biomedical Sciences Research Institute, Ulster University, C-TRIC Building, Altnagelvin Area Hospital, Glenshane Road, L/Derry, BT47 6SB UK; 2 Intelligent Systems Research Centre, Ulster University, L/Derry, BT48 7JL UK; 3 School of Psychology, Ulster University, Coleraine, BT52 1SA UK

**Keywords:** Depression, Microbiome

## Abstract

A growing body of evidence supports an important role for alterations in the brain-gut-microbiome axis in the aetiology of depression and other psychiatric disorders. The potential role of the oral microbiome in mental health has received little attention, even though it is one of the most diverse microbiomes in the body and oral dysbiosis has been linked to systemic diseases with an underlying inflammatory aetiology. This study examines the structure and composition of the salivary microbiome for the first time in young adults who met the DSM-IV criteria for depression (n = 40) and matched controls (n = 43) using 16S rRNA gene-based next generation sequencing. Subtle but significant differences in alpha and beta diversity of the salivary microbiome were observed, with clear separation of depressed and healthy control cohorts into distinct clusters. A total of 21 bacterial taxa were found to be differentially abundant in the depressed cohort, including increased *Neisseria* spp. and *Prevotella nigrescens*, while 19 taxa had a decreased abundance. In this preliminary study we have shown that the composition of the oral microbiome is associated with depression in young adults. Further studies are now warranted, particuarly investigations into whether such shifts play any role in the underling aetiology of depression.

## Introduction

Depression is a highly prevalent, complex mental health disorder characterised by a range of debilitating symptoms. Currently, as the leading cause of the global burden of disease, depression affects over 300 million people worldwide. In recent years, compelling evidence has emerged that dysfunction of the microbiota within the human gastrointestinal tract appears to play a key role in the pathophysiology of major depression via a bi-directional communication network known as the gut-brain axis^1^. Comparison of the microbiota composition of the gut in individuals with depression versus healthy individuals has revealed that, in general, depression is associated with a dysbiosis and reductions in both bacterial diversity and abundance^2^.

Along with the gut, the oral microbiome is also one of the most diverse microbiomes in the human body and similarly plays an important role in health and disease^3^. The mouth is highly vascularized and bacteraemia due to bacterial translocation across the epithelial mucosa is an everyday event^4^. This ‘mobile microbiome’ has the potential to cause metastatic infection, injury and inflammation^5^. Although alterations in the oral microbiotia are associated with local disease, most commonally caries and periodontal disease^6^, the latter may also represent a risk for other conditions^7^ including cancer^12^, cardiovascular and inflammatory bowel disease^8^ and rheumatoid arthritis (RA); in the latter case, markers associated with both RA risk and therapeutic response^9^ have been identified.

Links between poor oral health, neurological disorders, such as Alzheimer’s disease, and depression have been described ^6,10,11^; however, in such cases, disentangling cause from consequence is not straightforward. While the role of the oral microbiome in depression and anxiety has received little attention, two very recent investigations have reported changes in the composition of the salivary microbiome with depressive-like symptoms. Differential abundance of specific bacterial taxa, including *Spirochaetaceae, Actinomyces*, *Treponema*, *Fusobacterium and Leptotrichia* spp. were found to be associated with severity of depressive and anxiety symptoms in adolescents^12^. In addition, the overall composition of the oral microbiome and diurnal patterns of relative bacterial taxa abundance differed as a function of psychological distress and affective state in a non-clincal adult population^13^. Collectively, this data highlights the potential of the human oral microbiome as a source of novel diagnostic and therapeutic response biomarkers for mood and anxiety disorders. It also opens up new opportunities to investigate whether changes or shifts observed in oral microbial composition may directly contribute to the aetiology of mental disorders.

In the current study, we used bacterial 16S ribosomal RNA (16S rRNA) gene-based next generation sequencing (NGS) to profile the bacterial composition of saliva in young adults with depression versus healthy matched controls, and evaluate if changes to the structure and composition of the oral microbiota were associated with depression.

## Materials and methods

### Ethics

Ethical approval for this study was obtained from Ulster University Research Ethics Committee (REC/15/0004). All methods were performed in accordance with the relevant guidelines and regulations. Informed consent was obtained from all participants enrolled to the study.

### Study design

The Ulster University Student Wellbeing Study (UUSWS) was conducted as part of the WHO World Mental Health International College Student Project (WMH-ICS). First year students were recruited during registration where they gave written consent, provided a saliva sample, and were given a unique, anonymous number to complete an online mental health survey clinically validated against the Diagnostic and Statistical Manual of Mental Disorders IV (DSM-IV)^14^.

### Survey responses

The survey instrument was adapted from the WMH Composite International Diagnostic Interview (CIDI) version 3.0^15^. Life-time depression is determined based on the response to seven questions (Likert scale) corresponding to DSM-IV criteria for depression. To calculate lifetime major depressive disorder (LT-MDE), the first six symptoms/questions were recoded to; 4 = “all or most of the time” and 0 = ’none of the time’, and summed. If at least one of the first four symptoms was “all or most of the time” and the sum of all six symptoms was at least 15 then participants met the criteria for depression.

### Case selection

Cases (*n* = 40) were selected from particpants who met the criteria for LT-MDE. Healthy controls were individuals with no history of mental health problems (n = 43), closely matched to cases by age and gender and, where possible, by smoking status (Table 1). There was no significant difference in age (p = 0.16) or gender composition (p = 0.8) between the case and control groups. Gender and smoking were all included as potential confounders for microbiome composition in downstream analyses.

**Table 1:** Sample demographics based on participant response to CIDI depression section.

Demographics	Control (n = 43)	Depression (n = 40)
Age (mean)	20.4	21.8
(Range ± SD)	(18–36 ± 3.9)	(18–38 ± 5.1)
Gender		
Male (%)	13 (30.2)	11 (27.5)
Female (%)	30 (69.8)	29 (72.5)
Smoking status		
Smoker (%)	9 (20.9)	18 (45)
Non-smoker (%)	34 (79.1)	22 (55)
Depression		
Lifetime MDE	0 (0.0)	40 (100.0)
12-month MDE	0 (0.0)	39 (97.5)
Age of Onset, mean (Range ± SD)	–	14.4 (6–32 ± 4.1)
No. years of Life MDE	–	6.3 (1–21 ± 5.3)
Months of MDE in past year	–	6.9 (1–12 ± 4.1)

### Sample collection, DNA extraction, library preparation, and sequencing

Saliva samples (passive drool) were collected using Oragene OG-500 kits (DNA Genotek, Ontario Canada), enabling self-collection and stabilisation of DNA at room temperature. Participants were not to comsume any food or drink, except water, for at least 30 min prior to sample collection. Microbial DNA was extracted using the MasterPure™ DNA Purification Kit with Ready-Lyse™ Lysozyme (Epicentre, Madison, US), according to the manufacturer’s instructions. Samples were then sent to The Forsyth Institute, Cambridge, MA, USA for 16S rRNA gene-based NGS. PCR amplification of 10-50 ng of sample DNA was performed using 16S universal primers; V3–V4 341F (5’-CCTACGGGAGGCAGCAG-3’) and 806R (5′-GGACTACNVGGGTWTCTAAT-3′) primers. The amplicon products were purified using Solid Phase Reversible Immobilization with AMPure beads, and 100 ng of each amplicon library was pooled, gel-purified, and quantified using a bioanalyser. Finally, 12 pM of the library mixture was then spiked with 20% PhiX (Illumina, San Diego, CA), and sequenced on Illumina MiSeq, using a modified protocol from Caporaso et al^16^.

### Bioinformatic processing

Microbiome analysis was performed using QIIME 2 2019.07^17^. Paired-end sequence data generated with the Earth Microbiome Project amplicon sequence protocol^18^ were demultiplexed using the q2-demux plugin. Sequence reads were quality filtered, merged, and denoised using the DADA2 (v2018.11.0) algorithm^19^. Default parameters for demultiplexing (minimum phred score 33) and denoising were used except forward and reverse sequence truncation length (240 and 225, respectively). All amplicon sequence variants (ASVs) were aligned with mafft^20^ (via q2‐alignment) and used to infer phylogenies with fasttree2^21^ (via q2‐phylogeny). Taxonomy was assigned to ASVs using the q2‐feature‐classifier^22^ classify‐learn naïve Bayes taxonomy classifier against the Human Oral Microbiome v15.22 database^23^. Sequences (n = 329,683) that failed to resolve to at least phyla level were discarded (5.8% of 5,350,167 joined sequences). The processed data was imported into phyloseq v1.28.0^24^ for analysis. Alpha-diversity metrics; Abundance Coverage Estimator, Shannon, and Inverse Simpson^25^, beta diversity metrics (Bray–Curtis dissimilarity), and canonical correspondence analysis (CCA) were conducted using phyloseq.

### Statistical analysis

To identify common bacterial taxa associated with either healthy controls or depressed subjects we performed statistical analysis only on taxa present in at least 5% of samples (i.e. we filtered taxa by minimum prevalence across samples). The statistical analysis was performed with DESeq2 (v1.24.0)^26^ using a Wald test and Benjamini-Hochberg (FDR) correction^27^; statistical significance was determined at the 5% level. When performing statistical tests to identify differences in depressed subjects, smoking and gender were controlled for by including them as terms in the regression models.

## Results

### Species diversity of salivary samples differs between depression and healthy cohorts.

**Figure 1:**
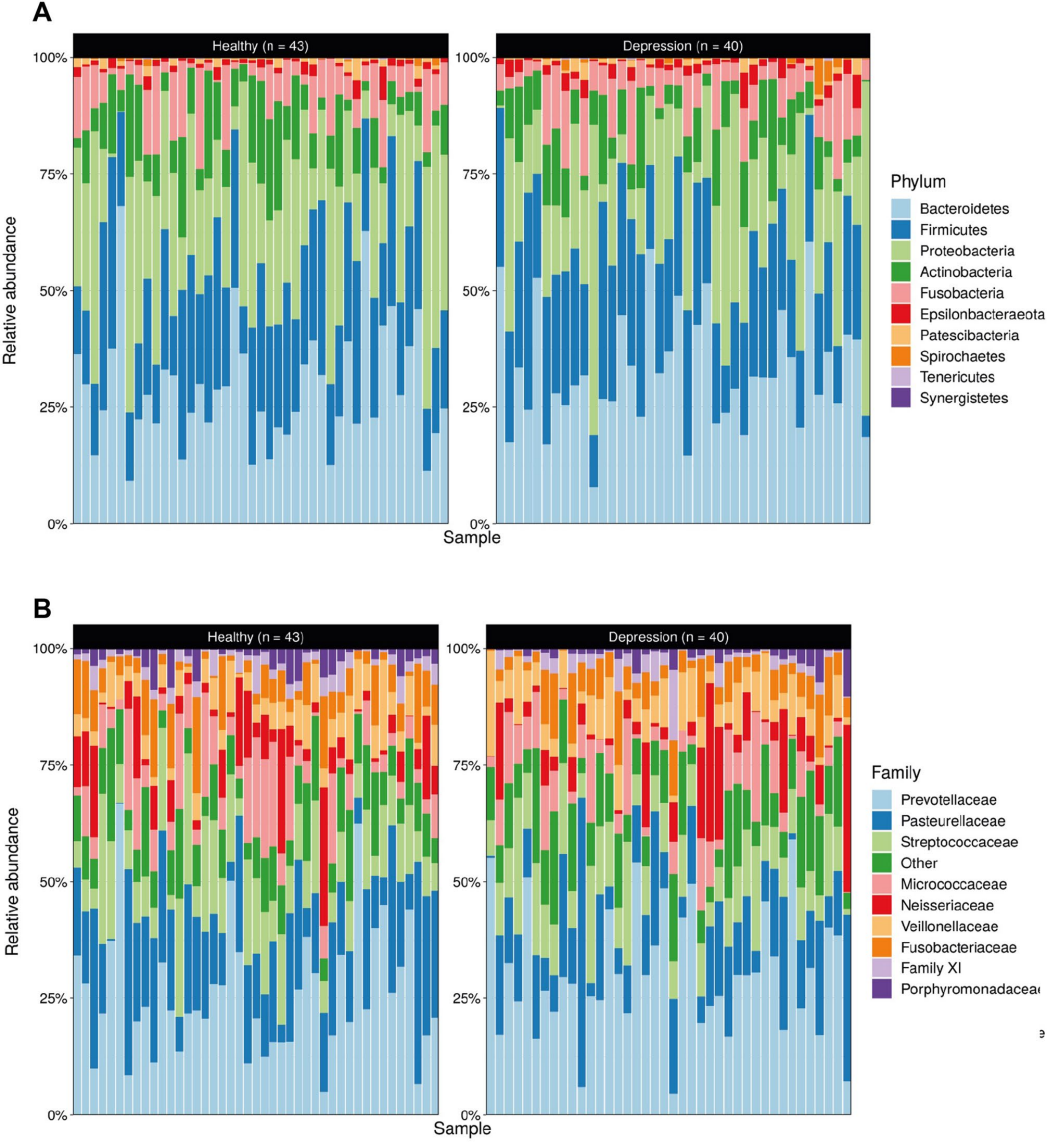
The overall composition of the oral microbiome matches previous reports in the literature: Community composition shown by relative abundance of prevalent (**A**) phyla and (**B**) families. Taxa present in fewer that 5% of samples and with a relative abundance smaller than 0.1% were removed.

Sequencing the V3–V4 regions of the 16S rRNA gene generated approximately 12.5 million sequences (median ± MAD): ~ 66,000 ± 28,000 sequence reads per subject, and the denoised dataset contained 3613 unique sequences covering 10 phyla, 19 classes, 42 orders, 75 families, 144 genera, and 181 identified species. The dominant phyla present in the salivary microbiome across both cohorts were *Bacteroidetes* (29.6 ± 11.8%), *Firmicutes* (24.5 ± 9.3%), and *Proteobacteria* (21.2 ± 9.3%) (Fig. 1A). The most prevalent families in the salivary microbiome for all subjects were *Prevotellaceae* (26.9 ± 10.8%), *Pasteurellaceae* (16.4 ± 10.3%), and *Streptococcaceae* (9.9 ± 5.3%) (Fig. 1B).

### The structure of the depressed microbiome in individuals with depression differs from control subjects

**Figure 2:**
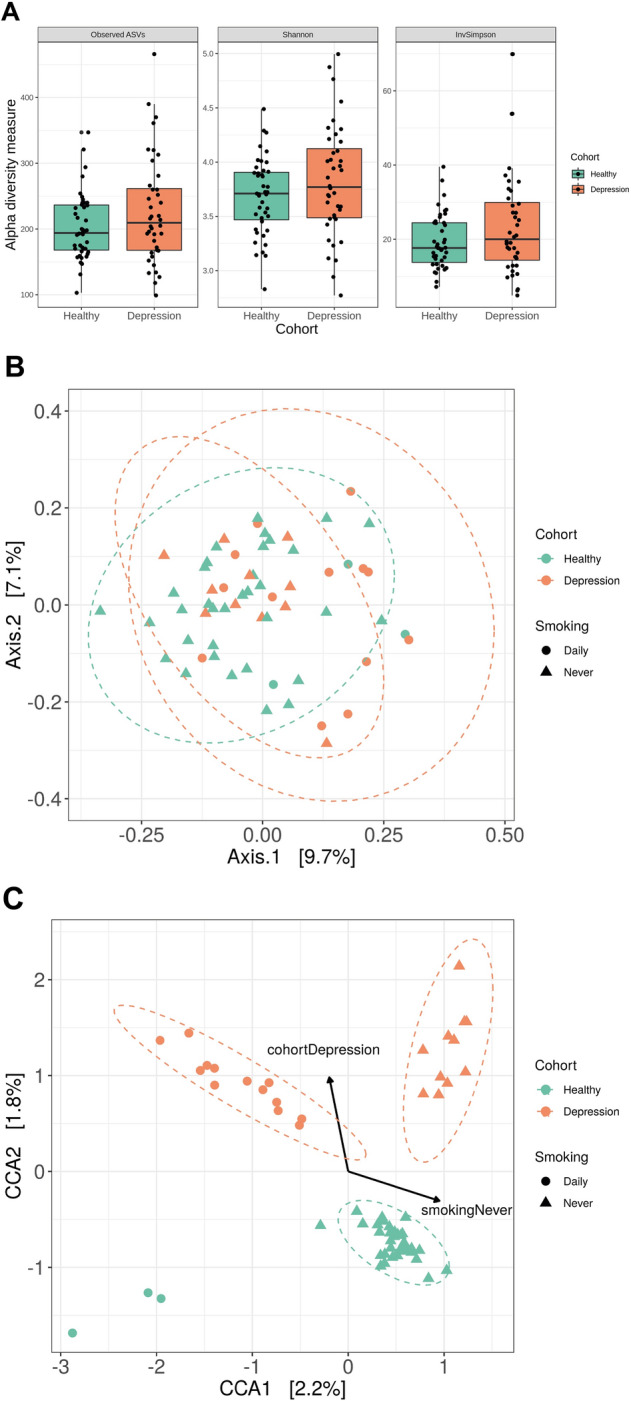
The structure of the oral microbiome in subjects with depression is subtly different compared with controls: (**A**) Shannon diversity is significantly more variable in the depressed cohort (F-test; p < 0.05). However, mean alpha diversity was not significantly different across cohorts for any alpha diversity indices (**B**) Unconstrained analysis of microbiome structure (PCoA) shows no significant clustering between cohorts (**C**) Constrained analysis of microbiome structure shows significant clustering between cohorts. Canonical Correspondence Analysis (CCA) relates samples (dots) to significant environmental variables (lines). The variation that can be explained by each axis is significant (cca anova; smoking p = 0.001; depression p = 0.002). The normal data ellipses (also known as concentration ellipses) serve to highlight clusters of samples.

The structure of the salivary microbiome was investigated by estimating the local diversity (α-diversity) of samples (Fig. 2A). To estimate α-diversity, we used the number of observed taxa, and the Inverse Simpson and Shannon diversity indexes. Shannon diversity was significantly more variable in the depressed cohort (F-test; p < 0.05), although mean alpha diversity was not significantly different across cohorts for any diversity indices. Smoking is highly prevalent amongst mental health populations compared to the general population so it was not possible to completely match for smoking and, as a consequence, there were significantly more current smokers in the depressed group (p = 0.016). CCA was used to test and visualise the effect of depression and smoking on the structure of the oral microbiota (Fig. 2B,C). The structure of the oral microbiome differed significantly between the depressed and control cohorts (anova.cca; p = 0.002), which clustered into distinct groups. As expected, smoking also significantly altered microbiome composition (anova.cca; p = 0.001) (Fig. 2C).The first canonical axis displays a negative correlation with daily smoking (anova.cca; p = 0.001), while the second was positively correlated with depression (anova.cca; p = 0.002). However, there was no interaction between smoking and cohort status, indicating that the separation of the depressed and control groups is independent of smoking.

### Differential abundance of specific bacterial taxa in the salivary microbiome of individuals with depression

**Figure 3:**
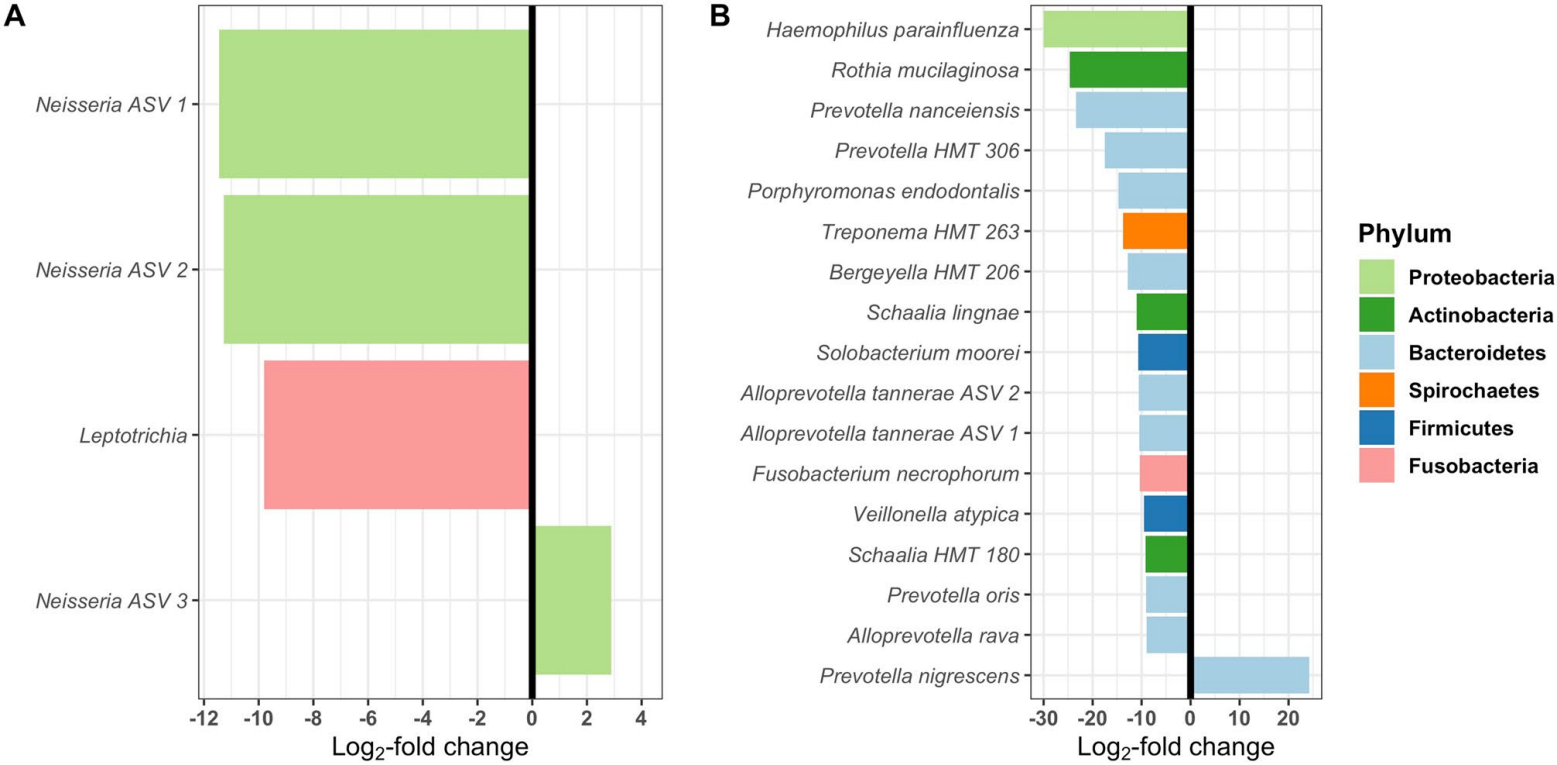
The abundance of amplicon sequence variants is significantly different in the depressed cohort: Differential abundance analysis identified 21 ASVs (that cover 13 genera and 6 phyla) that had a significantly altered abundance in the depressed cohort compared with healthy controls (padj < 0.05). (**A**) Differential abundance in 4 ASVs resolving to highest taxonomic level genus; (**B**) Differential abundance in ASVs with species level resolution.

Differential abundance testing of prevalent ASVs found that 21 bacterial taxa were differentially abundant in the depressed cohort relative to the controls. Of these, four ASVs resolved only to the genera level (Fig. 3A), and the remaining 17 were matched to bacterial species (Fig. 3B). From these sequence variants, two were significantly more abundant in depressed subjects, and 19 were significantly less abundant in depressed subjects. Plotting these 21 bacterial taxa shows clear differences in bacterial abundance between control and depressed groups (Fig. 3). *Prevotella nigrescens* (Wald test; p < 0.001) and *Neisseria* genera (Wald test; p = 0.02) were significantly more abundant in the depressed cohort. ASVs with unique sequences that matched to the same taxonomic group were given arbitrary identifiers to distinguish between them. ASVs in the genera *Prevotella*, *Haemophilus*, *Rothia*, *Treponema*, *Schaalia*, *Neisseria*, *Solobacterium*, *Lepotrichia*, *Fusobacterium*, and *Veillonella* were less abundant in the depressed cohort (Table 2).

**Table 2:** Statistically significant log2-fold change differences in the abundance of salivary bacterial taxa between depressed and healthy subjects.

Genus	Species	log2FoldChange	padj
*Haemophilus*	parainfluenzae	− 30	< 0.001
*Rothia*	mucilaginosa	− 24.71	< 0.001
*Prevotella*	*nanceiensis*	− 23.37	< 0.001
*Prevotella 7*	*HMT 306*	− 17.54	< 0.001
*Porphyromonas*	*endodontalis*	− 14.73	< 0.001
*Treponema 2*	*HMT 263*	− 13.78	< 0.001
*Bergeyella*	*HMT 206*	− 12.82	< 0.001
*Neisseria ASV 1*		− 11.44	< 0.001
*Neisseria ASV 2*		− 11.28	0.01
*Schaalia*	lignae	− 10.97	< 0.001
*Solobacterium*	moorei	− 10.65	< 0.001
*Alloprevotella*	*tannerae ASV 1*	− 10.56	0.01
*Alloprevotella*	*tannerae ASV 2*	− 10.49	< 0.001
*Fusobacterium*	*necrophorum*	− 10.34	0.01
*Leptotrichia*		− 9.81	0.01
Veillonella	atypica	− 9.47	0.05
*Schaalia*	HMT 180	− 9.23	< 0.001
*Prevotella*	*oris*	− 9.04	< 0.001
*Alloprevotella*	*rava*	− 9.02	0.01
*Neisseria ASV 3*		2.89	0.02
*Prevotella*	*nigrescens*	24.17	< 0.001

## Discussion

Given the well described body of evidence to support the critical role of the gut microbiome in mood disorders, in conjunction with the significant overlap that exists between the taxa in the gut and oral sites, we hypothesised that depression would also be associated with measurable changes in the oral microbiome. Here we report for the first time subtle changes in salivary microbiome structure and composition in adults with depression compared to controls. While there was no significant difference in α-diversity between control and depressed cohorts, significant differences in taxonomic abundance profiles (β-diversity) were identified enabling clear discrimination between the healthy and depressed cohort based on Bray–Curtis dissimilarities. At lower taxonomic levels, several genus and species differences were evident between the two cohorts, and we identified significant variation in 21 ASVs that cover 13 genera and six phyla, with 19 ASVs decreased and two increased in the depression cohort.

There is strong evidence from both preclinical animal models and clinical studies that depression is associated with the composition of the gut microbiome, including altered diversity and differential abundance of certain bacterial taxa^2,28,29^. Our data now adds to these previous results and suggests that depression also confers apparent and detectable changes in the salivary microbiome. Of the 21 bacterial taxa that were differentially abundant in the oral cavity, the majority (n = 19) were decreased in individuals with depression compared to controls, similar to previous reports with the gut microbiome^2^. While microbiome composition is site-specific^30^, there is evidence to indicate a degree of overlap and crosstalk between the oral and gut microbiomes. Oral microbiota are enriched within different gastrotestinal locations in individuals with treatment-naive microbiome in new-onset Crohn's disease (CD)^31^, suggesting that oral bacteria may colonise the gut and contribute to chronic inflammatory disease^32^. It is also very probable that microbes and their metabolites in the oral cavity may translocate or leak thorough a compromised blood–brain barrier, leading to neuroinflammation, an important feature in the aetiology of depression^33^.

Signs of oral dysbiosis were evident in our depressed cohort with ASVs corresponding to *Prevotella nigrescens,* a bacterial species previously linked to periodontitis^34^ and Th17 immune responses *in-vivo*^35^, demonstrating the most increased abundance in depression. While periodontal disease and depression share a number of environmental risk factors such as age, low socioeconomic status, smoking and alcohol consumption, sleep deprivation^36^ and stress^37,38^, predisposition to chronic and aggressive periodontitis also shares common genetic polymorphisms with depression in relation to the genes for BDNF, CXCL10 and 5HTT^57–59^. Inflammation plays a key role in both periodontitis^39^ and depression, and the presence and abundance of specific microbiota within the oral cavity that could contribute to both periododontitis and depression through a common host inflammatory response is highly probable. On this basis, the well described anti-inflammatory effects of antidepressants may help explain, at least in part, their efficacious effects in this context^40^ .

Strikingly, we observed a widespread reduction in several oral taxa in the depressed cohort versus controls, including known commensals. The largest difference was found with *Heamophilus parainfluanzae*, a common species found throughout the oral cavity which has anti-proliferative effects against cancereous cells but can also behave as an opportunistic pathogen^41,42^. *Rothia mucilaginosa* is also a common commensal in the oral cavity and produces enterobactin that reduces the growth of certain strains of cariogenic *Streptococcus mutans* and pathogenic strains of *Staphylococcus aureus*^43^. Reductions in this species, as observed in the depressed cohort, are associated with oral dysbiosis. Furthermore, *Schaalia lingnae*, formerly known as *Actinomyces lingnae*, has been identified as one of the core microbiota associated with a healthy oral cavity ^44^ and was found to be decreased in individuals with depression .

We can speculate that the lower abundance observed amongst organisms considered part of the normal microbiota may predispose to a more pathogenic or inflammatory microbial composition within the oral site of depressed subjects. In particular, lower levels of actinomyces have previously been linked with high anxiety and cortisol levels in adolescents^12^ and may be a marker of hyperactivation of the hypothalamic–pituitary–adrenal (HPA) axis, also common in the pathophysiology of depression. A number of taxa in the genus *Prevotella* have also been negatively associated with depression and psychological distress ^13^ and *Haemophilus* and *Neisseria* taxa are also depleted in the oral microbome of individuals with rheumatoid arthritis^9^, possibly indicative of an inflammatory state. It is, however, important to note that we did also observe a higher abundance of some species in the healthy cohort, including *Solobacter moorei*, *Alloprevotella tannerae and Porphyroomonas endodontalis*, that have been previously described in the context of halitotsis and periodontal disease^45–47^.

Yet, despite such observations, our understanding of the specific role of these and indeed other oral microbes in human health and disease remains poor, and it is unclear of the extent to which specific lineages with a heightened capacity to cause disease exist alongside strains of the same species that are more positively associated with health; this has been observed with other human commensal bacteria^48^. Such intraspecies differences can complicate interpretation of microbiome changes in health and disease, alongside complex interaction networks between taxa in disease states that we do not currently understand.

Smoking impacts directly and indirectly on oral bacteria^49^. In this sample set, a high portion of individuals with severe depression reported daily or occasional smoking, in comparison to a very low prevalence of smoking in healthy individuals. During sample selection, priority was placed on depression, with smoking status matched where possible. In our cohort, both depression and smoking significantly altered the microbial community composition in saliva as would be expected. The effects observed, however, did not appear to overlap and altered the microbiome in different ways based on the separation observed following CCA analysis. Furthermore, differential abundance in individual taxa were identified after controlling for smoking status. As a result, the effects of depression observed here were independent and not an artefact of smoking status.

Oral health and hygiene habits also impact on the oral microbiome. In this study, we did not collect data on oral health and have not controlled for this variable in the current report. There is a documented association between depression and poor oral health^50^ but the relationship is complex and while depression may lead to poor oral health in some cases, in others a lack of personal care may precede depression^51^. It is possible that the differences in the oral microbiome that we observed are not directly attributable to depression in all cases but a secondary consequence of poor oral health. It is also plausible that poor oral hygiene may be a precursor to poor overall health and systemic inflammation, a risk factor for depression. This link has received considerable attention in the context of cardiovascular disease but not been extensively studied to date for mental health. As noted above, bacterial species linked to periodontal disease have been found at higher levels in both healthy (*Solobacter moorei* and *Alloprevotella tannerae*) and depressed individuals (*Prevotella nigrescens*) so the relationship between oral health, depression and changes in the oral microbiome is complex and will require much further investigation.

Host bevahiours including dietary factors such as sugar intake can also alter the oral microbiome. Eating behaviour associated with depression can include consuming less food eating more or no change, and the composition of an individual’s diet will be highly variable^52^. Furthermore, the diet of the control cohort will naturally vary too, but as no information on food intake was recorded in the present we cannot determine the possible impact of diet on the differences observed.

Despite these limitations, in the present study we describe significant alterations in the overall bacterial composition and in abundance of specific bacterial taxa in the salivary microbiome in adults with depression compared to healthy controls. This work highlights the need for additional research into the potential role of the oral microbiome in mental health disorders to improve our understanding of disease pathogenesis, possibly leading to novel diagnostic targets and early intervention strategies.
